# A computerized neuropsychological test battery designed for idiopathic normal pressure hydrocephalus

**DOI:** 10.1186/2045-8118-11-22

**Published:** 2014-09-25

**Authors:** Anders Behrens, Anders Eklund, Eva Elgh, Cynthia Smith, Michael A Williams, Jan Malm

**Affiliations:** 1 Blekinge Centre of Competence, Blekinge Hospital Karlskrona, Karlskrona, Sweden; 2 Department of Clinical Neuroscience, Umeå University, Umeå, Sweden; 3 Centre for Biomedical Engineering and Physics, Umeå University, Umeå, Sweden; 4 Department of Radiation Science, Umeå University, Umeå, Sweden; 5 Department of Clinical Sciences, Psychiatry, Umeå University, Umeå, Sweden; 6 The Sandra and Malcolm Berman Brain & Spine Institute, Sinai Hospital of Baltimore, Baltimore, MD, USA; 7 Adult Hydrocephalus Center, Sinai Hospital of Baltimore, Baltimore, MD, USA; 8 Department of Neurology, Sinai Hospital of Baltimore, Baltimore, MD, USA; 9 Division of Neuropsychology, Sinai Hospital of Baltimore, Baltimore, MD, USA

**Keywords:** Dementia, Hydrocephalus, Normal pressure, Neuropsychological tests, Neuropsychology, Reliability and validity, Software

## Abstract

**Background:**

A tool for standardized and repeated neuropsychological assessments in patients with idiopathic normal pressure hydrocephalus (INPH) is needed. The objective of this study was to develop a computerized neuropsychological test battery designed for INPH and to evaluate its reliability, validity and patient’s ability to complete the tests.

**Methods:**

Based on a structured review of the literature on neuropsychological testing in INPH, the eight tests most sensitive to the INPH cognitive profile were implemented in a computerized format. The Geriatric Depression Scale (GDS) was also included. Tests were presented on a touch-screen monitor, with animated instructions and speaker sound. The battery was evaluated with the following cohorts: A. Test-retest reliability, 44 healthy elderly; B. Validity against standard pen and pencil testing, 28 patients with various cognitive impairments; C. Ability to complete test battery, defined as completion of at least seven of the eight tests, 40 investigated for INPH.

**Results:**

A. All except the figure copy test showed good test-retest reliability, r = 0.67-0.90; B. A high correlation was seen between conventional and computerized tests (r = 0.66-0.85) except for delayed recognition and figure copy task; C. Seventy-eight percent completed the computerized battery; Patients diagnosed with INPH (n = 26) performed worse on all tests, including depression score, compared to healthy controls.

**Conclusions:**

A new computerized neuropsychological test battery designed for patients with communicating hydrocephalus and INPH was introduced. Its reliability, validity for general cognitive impairment and completion rate for INPH was promising. After exclusion of the figure copy task, the battery is ready for clinical evaluation and as a next step we suggest validation for INPH and a comparison before and after shunt surgery.

**Trial registration:**

ClinicalTrials.org NCT01265251.

## Background

Cognitive impairment is a cardinal feature of idiopathic normal pressure hydrocephalus (INPH) and neuropsychological testing and grading are important for the diagnosis [1]. Patients show impairment in several domains, including memory, attention, executive functions, manual dexterity, psychomotor speed, and visuo-constructive ability [2-13]. It has also been demonstrated that some of the cognitive domains improve after shunt surgery [2,14-19]. Pre-operative investigations of INPH most often include drainage of cerebrospinal fluid in small or large quantities, and changes in cognition could be used for predicting improvement after surgery [20]. After shunt surgery, neuropsychological evaluation may be used to assess if the patient is improved, but also to decide on up- or down-regulation of an adjustable shunt [21]. However, there is no standardized neuropsychological test battery specially adapted and validated for INPH.

Computerized neuropsychological testing in the elderly has been suggested to have advantages compared to the corresponding conventional tests [22]. It could be administered in a standardized format; for example, instructions could be given in exact the same way at each session. Timed scoring of tasks, to avoid ceiling effects, can be used in a way that is impossible with paper and pencil tests [22]. Using a computerized test version, neuropsychologists are still needed for interpreting the findings and for diagnostic purposes, but the computerized test procedure probably requires a less skilled examiner. Using a computerized test version, data collection and scoring are objective and automatic. Thus, a computerized test has the potential to be administered to large groups of patients at a low price.

Neuropsychological testing of the elderly and patients with cognitive impairment such as INPH could be a challenge. A computerized environment is an additional complicating factor and it is important to evaluate that the computer – patient interface in a new test battery works for patients with INPH, also for those not being familiar to computers.

The aim of this study was to perform a structured literature search and identify the most appropriate paper and pencil tests for INPH. These conventional tests were translated into a computerized test battery that was evaluated regarding test-retest reliability, normative data of healthy elderly, validity for patients with cognitive impairment and finally, completion rate in INPH patients.

## Methods

**Figure 1:**
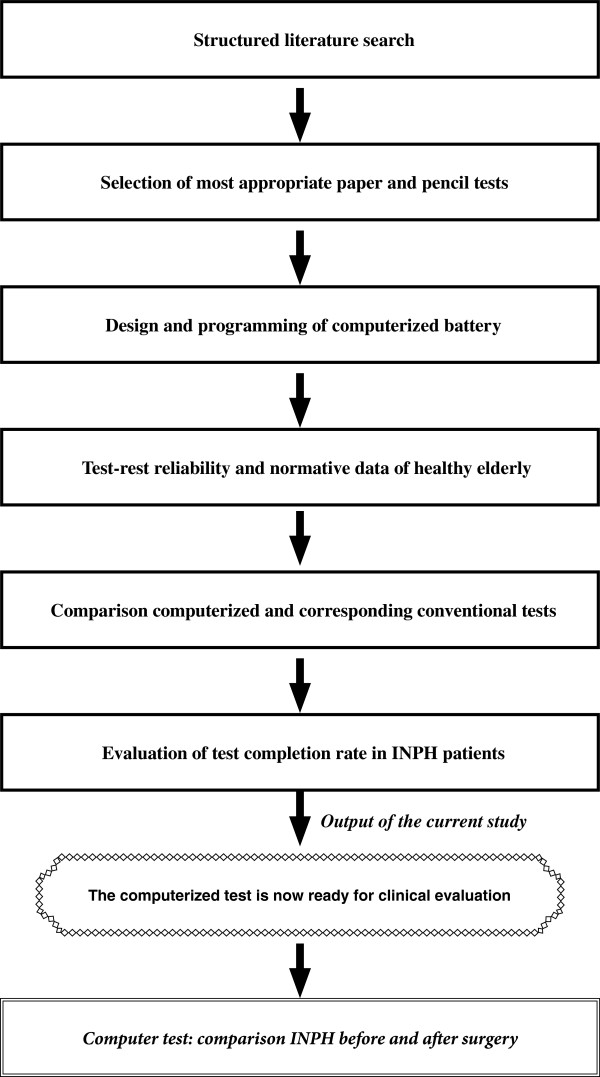
The research plan for this study.

The research plan for this prospective study is illustrated in Figure 1. In summary, design and programming of the computerized battery was based on a structured literature search. The evaluation of the test battery was divided into three parts: A. *test-retest reliability* to determine amount of measurement error in the computer tests; B. *validity* to ascertain that scores of the computerized tests co-vary with scores on their conventional paper and pencil test correlates, and; C. *ability to complete the test* for INPH patients.

### Participants

Recruitment and testing were done at Umeå University, Sweden. Table 1 summarizes demographical data for the study populations.

**Table 1:** **Characteristics of the study populations**^**a**^

	**Test-retest reliability (Healthy elderly)**	**Validity (Cognitive impairment)**	**Computer - patient (All hydrocephalus)**	**Computer - patient (INPH)**
Age, y Median (range)	69 (60-79)	71 (56-86)	72 (50-85)	69 (58-85)
Numbers (n)	44^b^	28	40	26^d^
Sex, % (M/F)	41/59	50/50	63/37	69/31
Education y, Median(range)	11.5 (6-22)	10 (6-15.5)	8 (6-20)	8.5 (6-20)
Computer knowledge^c^ % Yes	60	50	53	46
Color blind %	0	10.7	12.5	11.5
MMSE, Median (range)	>28	26 (20-30)	26 (18-30)	27 (20-30)
GDS, Median (range)	0 (0-6)	3 (0-10)	4 (0-19)	4 (0-19)

^a^MMSE = Mini Mental State Exam, GDS = Geriatric Depression Scale.

^b^Two tests (four finger tapping and ten word list learning) were redesigned during the study and only 26 of the 44 participants in the reliability group took the slightly modified battery.

^c^The subjects were asked “Do you have computer knowledge, yes or no”.

^d^Subgroup of the “computer - interface” group. Patients diagnosed with INPH.

**Figure 2:**
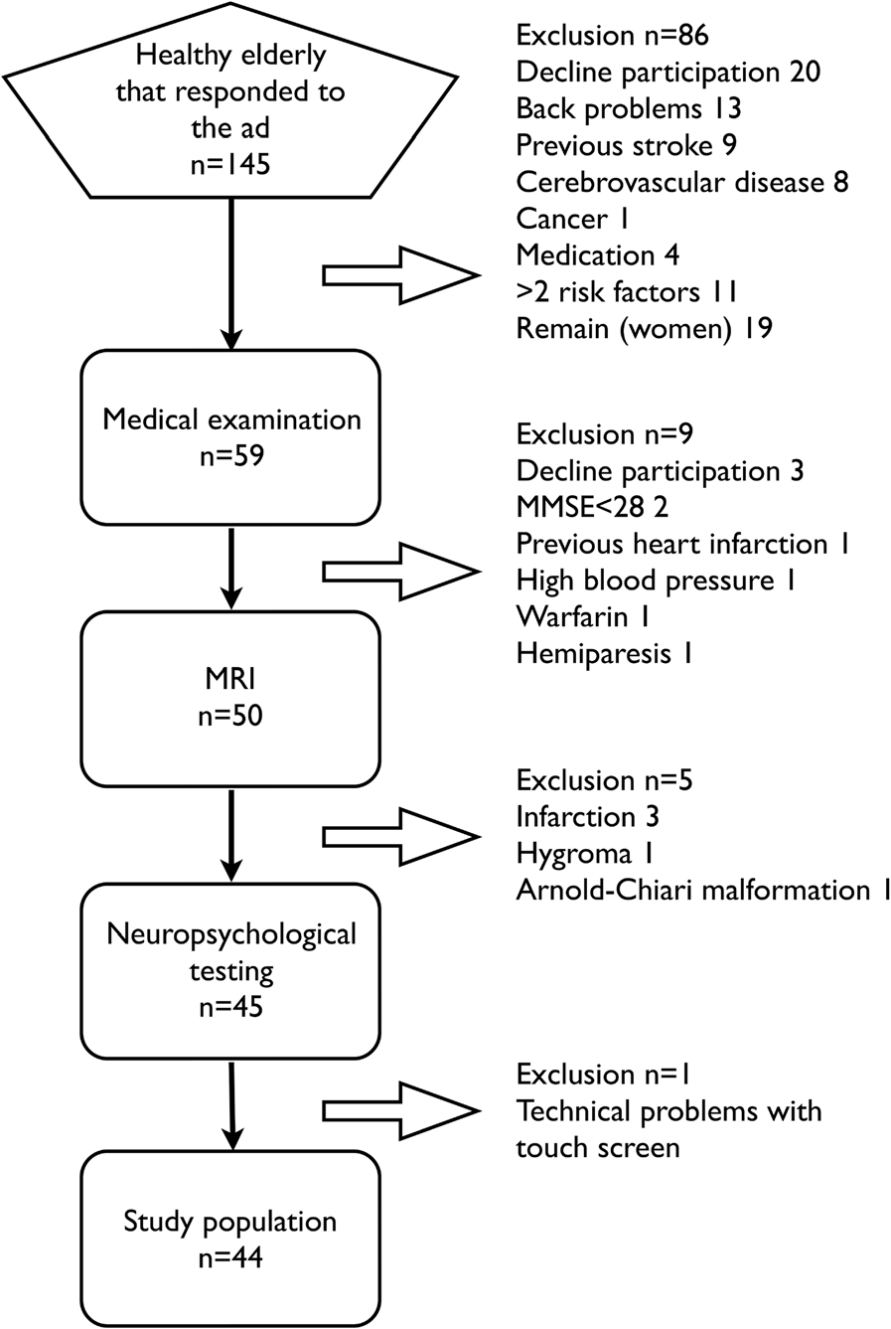
**Recruitment and exclusions in the reliability study.** The participants were confirmed healthy regarding medical history and clinical examination including ongoing medication, physical and neurological examinations, electrocardiography, blood pressure, body mass index, Mini-Mental State Examination (MMSE) and MRI. Exclusion criteria was disease of the nervous system, MMSE < 28, medications affecting nervous system (such as benzodiazepine or antidepressants), anticoagulants, ischemic heart disease, diabetes, and vascular risk factors (Two of either: hypertension, smoking or hyperlipidemia). The attempt to obtain equal numbers of men and women, resulted in the early exclusion of 19 women who responded to the ad i.e. the “Remain” group.

A. *Test-retest reliability.* An advertisement was placed in the local newspaper asking for healthy individuals (60-82 years old). The selection and definition of healthy elderly have previously been reported [23]. A flow diagram describing recruitment and reasons for exclusions is displayed in Figure 2. Forty-four healthy individuals were included.

B. *Validity.* Patients at the neurological ward were screened and could be included if the minimental state estimation (MMSE) was between 20 and 30 points. No exclusion criteria, except impaired motor function (e.g. palsy) were used. Thirty patients were screened. Two patients could not complete the battery and were excluded.

C. *Ability to complete the tests.* Forty patients referred because of communicating hydrocephalus (MRI verified) and clinical suspicion of INPH was asked to participate in this study. After the pre-operative evaluation, 26 patients fulfilled the criteria for INPH according to INPH guidelines [1].

The Regional Ethical Review Board (IRB) in Umeå approved the study and written informed consent was obtained from all participants. The study was prospective and is registered in ClinicalTrials.org no: NCT01265251.

### Test evaluation

The test was evaluated using three cohorts. To determine amount of measurement error attributable to the tests, the *test-retest reliability* method was used [24]. Correlation between repeated measurements provide an indirect measure of the amount error in a score. Repeated measurements also give an estimation of improvement due to the effect of practice. To limit the effect of day-to-day fluctuations in cognitive performance, healthy elderly were recruited. To obtain reference scores, this cohort was recruited to age match the typical INPH-patient.

*Validity (convergent validity)* is “the degree to which an instrument truly measures the construct it purports to measure” [24], i.e. the adapted conventional paper-and pen tests. The computer test and corresponding conventional neuropsychological tests were administered to the same patient the same day. Patients in this cohort were recruited to have a range of cognitive performance, and thus a favorable noise to signal ratio, giving detectable correlations between computerized and conventional tests. Tests used in the conventional paper and pen test battery were the Stroop congruent/incongruent words [25], Trail making test A and B [26], CERAD ten-words-list, delayed recall and recognition [27], CERAD figure copy test [27].

For assessing the ability of hydrocephalus patients to complete the tests, patients referred to our department for hydrocephalus evaluation were recruited. Patients were given the computerized test battery. All testing was performed before any CSF removal procedures.

### Neuropsychological test selection

A Pubmed literature search was performed using the terms “hydrocephalus AND (neuropsychology OR cognitive impairment OR neuropsychological testing OR dementia)”. If any relevant studies were found in the reference list of obtained papers, those were also included. Fifteen studies were identified fulfilling the following criteria: A, more than 20 patients enrolled; B, showing cognitive impairment or C, cognitive improvement after CSF diversion (tap test or CSF shunt). The tests used in these studies are presented in Table 2. Only tests that have been reported in at least two studies are displayed. Tests assessing different cognitive domains, and having the best evidence for evaluation of INPH patients were implemented. When alternatives existed, a shorter test was selected. Thus, a Ten-word-list test was used instead of the commonly used Rey Auditory and Verbal Learning Test (RAVLT). Some conventional tests did not lend themselves to computerization, such as the grooved pegboard and digit span tests, and were thus omitted.

**Table 2:** **Identified tests in the literature review**^**a**^

**Test**	**Number of studies**	**Reference**
Stroop test	7	[2,3,13,15,16,20,28]
Digit span	6	[3,11,15,28-30]
RAVLT	5	[2,3,15,20,28]
Line tracing	5	[2,11,12,20,28]
Trail making test A	5	[11,12,20,28,29]
Grooved pegboard	4	[3,11,15,28]
Trail making test B	4	[2,11,20,28]
Word fluency	4	[20,28-30]
Rey Osterrieth complex figure test	4	[2,20,28,30]
Simple reaction time^b^	3	[4,13,15]
Figure copy	3	[11,20,28]
Wechsler memory scale	3	[2,20,31]
Ten-words-list	2	[12,30]
Target reaction time	2	[3,15]
Tracks task	2	[3,15]
Symbol digit	2	[11,12]
Serial dotting^b^	2	[11,12]
Timed writing of the alphabet^b^	2	[20,28]
Cronholm-Molander memory test	2	[4,13]
Identical forms test	2	[4,32]
Bingleys memory test	2	[4,32]
Choice reaction time^b^	2	[13,32]
Finger tapping	2	[11,16]

^a^RAVLT = Rey Auditory Verbal Learning Test. ^b^Has not shown cognitive improvement after CSF diversion.

### Computerized tests

The computer program was developed in JAVA™ and Adobe Flash™ [33,34], and implemented on a Windows laptop (Lifebook A530, Fujitsu, Japan). Tests were presented in Swedish on a 17” touch screen monitor (L1730SF, LG Electronics, Seoul, South Korea), with animations and pre-recorded spoken instructions. A stylus (Pentopia T2300, Pilot, Tokyo, Japan) was used in all interactions with the touch-screen. Other means of input were via a microphone and a small numeric keyboard. Here follows a description of the computerized tests.

#### Two choice reaction test (attention)

A cross was presented in the middle of the screen with a button on either side. The subject was instructed to keep the stylus over the cross and then press one of the buttons as fast as possible when an arrow appeared that pointed to the button to be pressed. The arrow appeared after a random interval of 5 to 15 seconds. The reaction time was measured as the interval between the appearance of the arrow and the time the correct button was pushed. Median reaction time over 20 trials was used as the test score.

#### Trail making test A (psychomotor speed)

On the screen, 25 buttons marked with numbers (1-25) were displayed. The subject was asked to press buttons in consecutive order (1-2-3-etc) as fast as possible. Errors were indicated with pre-recorded verbal feedback, “Wrong, push another button”. Time to completion was measured and used as test score.

#### Trail making test B (executive function)

The subject was presented with 25 buttons marked with numbers (1-13) and letters (A-L) on the screen. Buttons were to be pressed in consecutive order by alternating between letters and digits (1-A-2-B-3-C…). Errors were indicated with pre-recorded verbal feedback. Time to completion was scored.

#### Stroop congruent colors (psychomotor speed)

The names of colors (red, green, yellow or blue) were displayed in text of a black color. Two buttons of different colors were displayed, one of which corresponded to the name of the color presented. The subject was asked to press the button of the color that corresponded to the name of the color presented. Reaction time was measured as the interval between word presentation and the time the correct button was pushed. After the last button was pressed, there was a delay of 2 seconds before the next word appeared. Median reaction time for 50 words was used as the test score.

#### Stroop incongruent colors (executive function)

The names of colors were displayed in text of a color that was not congruent with the name of the color (e.g., the word red was shown in blue text). Two buttons of different colors were displayed, one of which matched the color of the text presented. The subject was asked to press the button of the color that corresponded to the color of the text as quickly as possible. Reaction time was measured as the interval between the word presentation to the time the correct button was pushed. Median response time for 50 words was used as the test score. If the error rate was more than 50%, the test was regarded as failed.

#### Ten-word-list (memory and learning)

The subject was asked to remember 10 consecutive words. The words were randomly drawn from a pool of the 50 most common Swedish nouns [35]. Words were presented on screen simultaneously with a recording of an announcer reading the word aloud. Each word was presented for two seconds, with a delay of two seconds between words. After the words were presented, the subject was asked to repeat as many of the words as possible into a microphone and save the answers by pressing a button marked “done” on the screen. The same list of words was presented three times, with the words in different order. The test score was the sum of correctly remembered words over the three trials.

#### Delayed recall (memory and learning)

After approximately 20 minutes of distracter tasks, the subject was asked to repeat the 10 words from the list-learning task. The number of correctly recalled words was used as the score.

#### Delayed recognition (memory and learning)

The subject was asked to discriminate between 10 words from the list learning task and 10 distracter words that were drawn from the same pool of 50 words. Twenty words were presented consecutively and the subject was asked to press buttons on screen: “yes” if recognized and “no” if not. The test score was calculated as the number of correct responses minus errors.

#### Figure copy task (visuo-spatial ability)

The subject was asked to copy a cube presented on screen, by drawing with the stylus. The drawing was stored for later manual scoring. The main author manually graded the figures, after all tests had been completed, as “correct” or “incorrect”. The figure was regarded correct if the size was correct and all lines were present.

#### Four-finger tapping (manual dexterity)

The subject was required to tap on a small keyboard with the 2nd to 4th fingers of the dominant hand. The correct order of tapping was (digits) 2-3-4-5-4-3-2-3-4 etc. The computer gave auditory feedback with a high-pitched tone when a correct button was pressed and a lower pitched tone for an incorrect selection. The tapping was to be performed as quickly as possible and was repeated five times. Each set was 10 seconds with time to rest in between. The number of correct taps during each set was measured. The total number of correct taps for all five sets was the score.

#### Geriatric Depression Scale (GDS)

GDS is a short instrument intended to measure symptoms of depression in elderly patients [36]. A score above 5 (range 0-20) indicates depression. The instrument has shown good validity [37]. The questions were displayed on the screen, and the subject was asked to press buttons labeled “yes” or “no”.

### Procedure

All testing was administered in a closed, sound-attenuated testing room, with a supervisor attending. In those tested twice (validity and reliability), one of two investigators (a research nurse or AB) attended at each session. Among INPH patients, testing patient-computer interface and the ability to complete the test, the research nurse attended all sessions. The investigator (AB) who administered the conventional tests was trained and supervised by a neuropsychologist (EE). The investigators were blinded to any previous results. The investigators were instructed to answer questions about the tests, but not to help during testing. In order to make the patients familiar with the computer equipment, this investigation started with a simple introductory task requiring the participants to press buttons on the screen with a stylus. Each test was preceded by a practice test. There was automatic recorded verbal feedback if the task was misunderstood. Results were stored to disk, and a test report was automatically generated for each test session.

### Statistical methods

The Pearson correlation between test and retest was used as reliability estimate. For the figure copy test, the phi-correlation coefficient was used. When applicable, the standard error of measurement (SEm) was calculated as SEm = SD*sqrt(1-r), where SD, is the standard deviation of the test scores, and r the Pearson correlation between test and retest [24]. The SEm gives an error band around a single score, and a given score is approximately within the range ± 2*SEm with a confidence of 95%. Practice effects between test and retest scores were analyzed with the Wilcoxon signed-rank test when normality assumption was not met; otherwise paired T-tests were used. For the figure copy test the McNemar test was used. Multiple linear regression was used to explore influence of demographical data on test scores. Convergent validity was explored by Spearman correlations between related computer/conventional tests. Discriminant validity, the degree to which tests from different cognitive domains does not co-vary, was explored by spearman correlations between the different computerized tests. The performance of INPH patients was expressed as percentage of median performance of the healthy individuals. Scores from the three cohorts were compared with the Mann-Whitney U test. For the figure copy test the Chi-square test was used. Significance level for all statistical data was set to 0.05. All statistics were analyzed in SPSS (Version 20, SPSS, Chicago, IL, USA).

## Results

### Test-retest (healthy elderly)

Scores, reliability, standard error of measurement (SEm) and significance level for practice effects are displayed in Table 3. Most of the implemented tests show a good reliability (r = 0.7 - 0.9), and all, but the figure copy test showed test-retest reliability above 0.6. Improvement between test and retest was seen in 5 out of 10 tests. Demographical influence on scores was seen in gender (Choice reaction test, male gender -79 ms, *p* = 0.015), education (delayed recall -0.2 words/year, *p* = 0.016) and age (Stroop congruent words 7 ms/year *p* = 0.015; Stroop incongruent 25 ms/year *p* = 0.07; Trail making test A 1.0 sec/year, *p* = 0.032; Trail making test B 2.6 sec/year, *p* = 0.004; finger tapping -4.5 taps/year, *p* = 0.043; delayed recall -0.1 words/year, *p* = 0.049). Reported computer knowledge did not influence any of the test scores.

**Table 3:** **Results for the test-retest investigation**^**a**^

**Computer test**	**Test 1 N = 44 median (IQR) (Day 1)**	**Retest N = 44 median (IQR) (Day 7 - 65)**	**Difference, median (IQR)**	***p***	**SEm**	**Reliability**
**Two choice reaction [ms]**	737 (660 - 822)	735 (634 - 818)	−16 (-62 - 28)	0.06^b^	51	0.75
**Stroop congruent [ms]**	846 (790 - 924)	841 (787 - 919)	−3 (-53 - 23)	0.4^c^	56	0.74
**Stroop incongruent [ms]**	1073 (952 - 1371)	1021 (865 - 1258)	−93 (-184 - 21)	<0.01^c^	149	0.83
**Ten word list**^**e **^**#**	20 (17 - 22)	22.5 (19 - 24.25)	2 (0.5 - 3)	<0.001^b^	2.1	0.67
**Delayed recall**^**e **^**#**	6 (5 - 8)	7 (4.75 - 8)	0 (-1 - 1)	0.78 ^c^	1.2	0.74
**Delayed recognition**^**e **^**#**	9 (9 - 10)	9 (8 - 10)	0 (-1 - 1)	0.65 ^c^	0.9	0.70
**Trail making test A [s]**	39.9 (36.0 - 48.8)	38.2 (33.5 - 44.7)	−2.2 (-7.0 - 2.4)	< 0.05^c^	6.5	0.87
**Trail making test B [s]**	78.2 (63.6 - 99.2)	74.7 (62.0 - 93.1)	−5.9 (-14.5 - 2.7)	< 0.05^c^	14.3	0.83
**Figure copy task [%]**	90.9^f^	81.8^f^	N/A	0.22 ^d^	N/A	0.57
**Four finger tapping**^**e **^**#**	108 (97 -149)	123 (103 -173)	12 (3 - 22)	<0.001^c^	15.8	0.90

^a^IQR = Interquartile range; p = significance in score difference between test and retest; SEM = Standard Error of Measurement.

^b^Paired t-test.

^c^Wilcoxon signed-rank test.

^d^McNemar test.

^e^Tests redesigned during the study and only 26 of the 44 participants in the reliability group took the slightly modified battery.

^f^Percent who scored “correct”.

### Validity (patients with cognitive impairment)

Significant correlations between conventional and computerized measures were seen in all tests (r = 0.49-0.83), see correlations and raw scores in Table 4. Table 5 demonstrates correlations between the different subtests. Significant correlations are seen between tests in the same cognitive domain i.e. tests of psychomotor speed, memory and executive function. As well, correlations are seen between tests with a strong motor component e.g. the Stroop tests, the Trail making tests and the finger tapping test. A correlation was also seen between the delayed recognition and figure copy tests.

**Table 4:** Results from the validity investigation

**Computer test**	**Test result computer test N = 28 median (IQR)**	**Correlation between computerized and conventional tests N = 28**	***p***^**b**^
**Two choice reaction [ms]**	847 (743 - 1122)	^a^	^a^
**Stroop congruent [ms]**	967 (857 - 1439)	0.82	<0.001
**Stroop incongruent [ms]**	1365 (887 - 2066)	0.76	<0.001
**Ten word list #**	14 (11 - 18)	0.66	<0.001
**Delayed recall #**	4 (2 - 6)	0.72	<0.001
**Delayed recognition #**	8.5 (7 - 10)	0.49	<0.01
**Trail making test A [s]**	53 (39 - 76)	0.85	<0.001
**Trail making test B [s]**	113 (77 - 189)	0.83	<0.001
**Figure copy task [%]**	61	0.54	<0.01
**Four finger tapping #**	89 (64 - 112)	^a^	^a^

^a^No corresponding paper and pencil test exists.

^b^Significance of correlation coefficient.

**Table 5:** Correlation matrix of healthy individuals’ performance at first computer test session

**Tests**	**Two choice reaction**	**Stroop congruent**	**Stroop incongruent**	**TMT A**	**TMT B**	**10 word list**	**Delayed recall**	**Delayed recognition**	**4-Finger tapping**	**Figure copy task**
**Two choice reaction**	1									
**Stroop congruent**	0.63	1								
**Stroop incongruent**	0.4	0.43	1							
**Trail making A**	NS	0.45	0.46	1						
**Trail making B**	NS	NS	0.48	0.73	1					
**10 word list**	NS	NS	NS	NS	NS	1				
**Delayed recognition**	NS	NS	NS	NS	NS	0.61	1			
**Word recognition**	NS	NS	NS	NS	NS	0.42	0.44	1		
**4-Finger tapping**	NS	−0.49	−0.56	NS	−0.39	NS	NS	NS	1	
**Figure copy task**	NS	NS	NS	NS	NS	NS	NS	0.44	NS	1

NS = Non significant.

### Ability to complete the tests (computer – patient interface)

**Figure 3:**
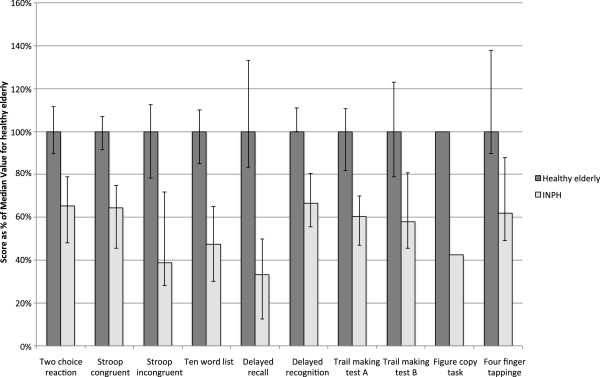
**Performance of patients with INPH, as percentages of median performance in healthy individuals.** Error bars indicate the interquartile range. To make impairments clear, results from tests with time scores were recalculated as units per time. Thus a lower score always mean impairment. The performance for the figure copy task is expressed as the ratio of correct response-ratios in the INPH and healthy groups. The INPH patients performed worse than healthy controls in all tests (Mann-Whitney, and Chi-square test for the figure copy task, *p* < 0.001).

The INPH group scored higher in the GDS (median = 4) than healthy individuals (median = 0) (Mann-Whitney, p < 0.001). Three patients did not complete the battery. They were 77, 79 and 85 years old and had MMSE scores of 23, 24 and 18. Two were too tired and therefore chose not to complete the battery, and one did not understand the instructions. Of all 40 patients, 31 (78%) completed the battery with a completion with at least seven out of eight tests. Ten patients (25%) failed to complete Trail making test B (needed help to complete the test) and 12 (30%) failed the Stroop incongruent words test (made >50% errors). Those who failed these two tests had lower scores on MMSE (Mann-Whitney, p = 0.005 and p = 0.015). Also, four patients did not perform the Four-finger tapping test as intended (only used the index finger). Figure 3 displays the results of the subgroup diagnosed with possible INPH, as proportion of the median performance of healthy individuals. Raw scores compared to healthy elderly are displayed in Table 6. The INPH patients performed worse than the healthy individuals on all tests. The interquartile score ranges were non-overlapping for all tests, but a slight overlap in the Trail making test B. Comparing the cognitive impaired patients of the validity group to healthy elderly, the cognitive impaired group performed significantly worse in Stroop congruent words, Ten word list, Trail making test A and B and the Figure copy tests (Mann-Whitney, and Chi-square test for the figure copy task, *p* < 0.05). Comparing INPH patients to cognitive impaired patients of the validity group, there was a trend that INPH patients performed worse in all tests. Significance was found in Stroop congruent words, Ten word list, Delayed recognition and Four finger tapping (Mann-Whitney, *p* < 0.05). There were more men, and more colorblind patients in the INPH group compared to healthy elderly (Chi-square test, *p* < 0.05 for both variables). No significant difference in age, education, preferred hand or computer knowledge was found. The single demographical variable that influenced any score in this group was education in the Stroop congruent words test (-122 ms/year, *p* = 0.025).

**Table 6:** **Test results from healthy elderly and INPH patients**^**a**^

**Computer test**	**First test healthy elderly N = 44 median (IQR)**	**INPH N = 26 median (IQR)**	***p***^**b**^
**Two choice reaction [ms]**	737 (660 - 822)	1130 (933 - 1534)	<0.001
**Stroop congruent [ms]**	846 (790 - 924)	1314 (1130 - 1857)	<0.001
**Stroop incongruent [ms]**	1073 (952 - 1371)	2764 (1494 - 3818)	<0.001
**Ten word list**^**c **^**#**	20 (17 - 22)	9.5 (6 - 13)	<0.001
**Delayed recall**^**c **^**#**	6 (5 - 8)	2 (0.75 - 3)	<0.001
**Delayed recognition**^**c **^**#**	9 (9 - 10)	6 (5 - 7.25)	<0.001
**Trail making test A [s]**	39.9 (36.0 - 48.8)	66 (57 - 85)	<0.001
**Trail making test B [s]**	78.2 (63.6 - 99.2)	135 (97 - 172)	<0.001
**Figure copy task [%]**	90.9^d^	38.5^d^	<0.001
**Four-finger tapping**^**c **^**#**	108 (97 -149)	67 (53 - 95)	<0.001

^a^IQR = Interquartile range; p = significance in score difference.

^b^Chi-square test for the figure copy task and Mann-Whitney U for all other tests.

^c^Tests redesigned during the study and only 26 of the 44 participants in the healthy elderly group took the slightly modified test.

^d^Percent who scored “correct”.

## Discussion

We have developed a novel computerized neuropsychological test battery customized for the evaluation of communicating hydrocephalus and INPH. Computerization makes test delivery and scoring standardized. The implemented test was easy to use, automated, and the administrator does not need special training. The battery takes 30-40 minutes to complete, and automatically delivers a printed report with scores and comparison to healthy elderly. Most tests showed good test-retest reliability and validity, and test completion rate was good for INPH patients. The new battery revealed that patients with INPH performed worse on all tests, including depression scoring, compared to healthy controls. The computerized test is now ready for clinical evaluation, however, the authors want to stress that this study was not designed to assess the ability of the battery to detect improvement after CSF removal or shunt surgery in INPH.

The translation of paper pen testing into computerized procedures may affect the reliability and validity of the test procedure [22,38]. An ideal neuropsychological test would have a high correlation with repeated measurements (i.e., test-retest reliability), indicating a low proportion of error in the test score. Most of the implemented tests show good to high reliability (r = 0.7 - 0.9). The exceptions are tests of memory and visuo-constructive ability. Reliability measures in the memory domain are typically relatively poor, and have been attributed to variable human performance [39]. However, refraining from measuring these abilities is not an option, as they are common patient complaints and important for describing typical features of the dementia in INPH. The poor reliability in the figure copy task is probably due to dichotomous data, where a small error has a large influence on the score (from pass to fail). This is also the only test where the scoring is based on judgment by the investigator. These drawbacks suggest leaving this test out in an updated version of the battery. Regarding the Ten-word memory test, the only test having reliability below 0.7 (r = 0.67), the correlation is influenced by the variability of the scores. The scores of healthy elderly show a relatively homogenous distribution and therefore the correlation does not necessarily reflect low accuracy in test scores. Another, more practical measure of reliability that is less affected by performance of group under study is the standard error of measurement (SEm) (Table 3), which gives an error band around a given score. The median performance of the INPH patients on the memory test is 9.5 remembered items. The true score for a patient with this performance would be within the range 5.3 - 13.7 items (median ±2*SEM). This range is with confidence lower than that of median healthy performance (20 items), implying satisfying reliability with regards to discriminating healthy from diseased. The ability to detect improvement after CSF diversion remains to be examined.

A common method to minimize the practice effects of a test is to use alternate forms. When designing the computer battery it was regarded practical to only have one form of each test. To limit the influence of the practice effect on the word list test, which is especially prone to practice effect, the program was designed to randomly draw 10 words from a pool of 50 words. Improvement between test and retest was seen in 5 of 10 subtests. Practice effects are influenced by age, retest interval and performance on the first test session [39]. This effect is important to notice when performing repeated testing in INPH, e.g., after shunt surgery or a tap test, where a mere practice effect can be taken for actual improvement. However, Solana *et al.* found no practice effects for selected subtests while performing repeated neuropsychological testing in INPH [40]. The reported test-retest improvement is calculated from healthy individuals, and is thus probably lower when testing in INPH patients. Also, the follow up time after shunt operation is typically three to six months. This time span would further limit the effect of practice [39]. Retest data on shorter time intervals, for instance before and after a short-term tap test, has to be evaluated in future studies.

Poor reliability of either the computer test or corresponding conventional test leads to poor validity. Conventional and computerized tests in the memory domain and Figure copy task show relatively low correlation. However, the correlations are in parity or better than other computerized batteries available [41,42]. The Four finger tapping test has no conventional test correlate. The test was previously studied during fMRI in our department, and was shown to improve after lumbar drainage in INPH-patients [16]. Thus, the test has validity in form of criterion validity. Divergent validity of the different subtests is demonstrated in Table 5. Not surprisingly there were correlations between tests in the same cognitive domain. There were also correlations between tests with a strong motor component. The use of a touch screen interface means that there is a motor component in most tests, which might be a problem for patients with severely impaired motor function; however, because the purpose of the battery is to assess performance at baseline in comparison to controls, and change from baseline in response to CSF drainage or shunt surgery, the test should accurately reflect any change in the combined effect of INPH on cognitive and motor processing speed. A comparable problem also exists with conventional paper and pen testing, e.g. in the Trail making test A or B. The computerized tests resemble their conventional paper and pen correlates, with exception of the Stroop tests, which had to be adapted for the touch screen format. The core of the test is the response conflict between acting on the text or the text color. In spite of different means of action, we believe that the executive core of the test is captured in the computer test, and that this is reflected in a longer response time in the test of incongruent colors compared to the congruent test. Also, the correlation between the computerized and conventional format of this test was 0.76.

Seventy-eight percent of the INPH patients completed the battery with one or none failed test. The ability to use the test in the intended patient group was thus good. The tests that were most commonly incomplete in the computerized battery were tests of executive functions (Trail making test B and Stroop incongruent words), which is a pattern seen in many forms of dementia. The percent incomplete tests is in parity with conventional neuropsychological testing in INPH, where in one study, the Stroop test was completed by 70% of the patients [43].

The INPH patients had significantly higher depression scores when compared to healthy individuals. Depression can impair episodic memory, processing speed and executive functions [44]. This underlines the importance of screening for depression when interpreting scores in dementia patients.

The potential for the different subtests to discriminate between healthy and INPH patients is demonstrated in Figure 3, which demonstrates impaired performance on all tests compared to healthy controls. Additionally, it is evident that the interquartile ranges for healthy and INPH patients are non-overlapping in all tests but the Trail making test B. Delayed recall being the most impaired test is in line with previous studies comparing healthy and INPH-patients [15]. There were significantly more men and colorblind patients in the INPH group. Male gender was associated with a faster response in the two choice reaction time test. Adjusting for this variable the result would still hold.

Recently, a new scale specifically designed for INPH was introduced [45]. The scale measures four domains, and neuropsychology as one. Even if the computerized scale presented in this study is not exactly the same, we consider us to have included similar tests. INPH scale included the Stroop test, the first part of the RAVLT and the pegboard test. Stroop test was included and the included 10-word list is similar in design as the RAVLT test. The Pegboard test is part of the INPH scale, but not included in the present battery. Instead, we have chosen the manual dexterity test that have been shown to improve after external lumbar drainage with a corresponding change in fMRI [16].

The implemented battery is a focused battery with tests chosen to be sensitive to the cognitive profile of INPH. The battery was never intended to be a diagnostic battery in the way that a neuropsychologist administers a battery. Therefore, its utility for differential diagnosis was not examined. It should be warranted that computerized testing will not provide the qualitative data, or interpretation of scores, as from a full neuropsychological evaluation. This requires a professional neuropsychologist. Although the test is fully automatic, for ethical reasons, the presence of personnel while testing is mandatory, as to clarify instructions or halt the test, if cannot be completed [22]. The battery is translated from Swedish to English and Danish. The software will remain free of charge and we have an ambition to translate it to more languages.

## Conclusions

A new computerized neuropsychological test battery designed for patients with communicating hydrocephalus and INPH was introduced. Its reliability, validity for general cognitive impairment and completion rate for INPH was promising. After exclusion of the figure copy task, the battery is ready for clinical evaluation and as a next step we suggest validation for INPH and a comparison before and after shunt surgery.

## Abbreviations

INPH: Idiopathic Normal Pressure Hydrocephalus; CSF: Cerebrospinal fluid; MMSE: Minimental state exam; SEM: Standard error of measurement; GDS: Geriatric depression scale; RAVLT: Rey auditory and verbal learning test.

## Competing interests

The authors declare that they have no competing interests.

## Authors’ contributions

All authors contributed to the conception and design of the study and drafting and revision of the manuscript. AB contributed to the implementation, data acquisition and analysis of the data. AE contributed to the analysis and interpretation, management and fund raising. EE, CS, MW and JM contributed to the analysis and interpretation of the data. JM contributed to management and fund raising. All authors have read and approved the final version of the manuscript.
